# Does Cognitive Dysfunction in Bipolar Disorder Qualify as a Diagnostic Intermediate Phenotype?—A Perspective Paper

**DOI:** 10.3389/fpsyt.2018.00490

**Published:** 2018-10-08

**Authors:** Lars Vedel Kessing, Kamilla Miskowiak

**Affiliations:** ^1^Copenhagen Affective Disorder Research Centre (CADIC), Psychiatric Centre Copenhagen, University Hospital of Copenhagen and University of Copenhagen, Copenhagen, Denmark; ^2^Department of Psychology, University of Copenhagen, Copenhagen, Denmark

**Keywords:** cognition, cognitive dysfunction, bipolar disorder, unipolar disorder, schizophrenia, intermediate phenotype, endophenotype

## Abstract

The present perspective paper addresses and discusses whether cognitive dysfunction in bipolar disorder qualifies as a diagnostic intermediate phenotype using the Robin and Guze criteria of diagnostic validity. The paper reviews current data within (1) delineation of the clinical intermediate phenotype, (2) associations of the intermediate phenotype with para-clinical data such as brain imaging and blood-based data, (3) associations to family history / genetics, (4) characteristics during long-term follow-up, and (5) treatment effects on cognition. In this way, the paper identifies knowledge gaps and suggests recommendations for future research within each of the five areas. Based on the current state of knowledge, we conclude that cognitive dysfunction does not qualify as a diagnostic intermediate phenotype or endophenotype for bipolar disorder, although promising new evidence points to emotion and reward processing abnormalities as possible putative endophenotypes.

Cognitive dysfunction in bipolar disorder is a core illness symptom that has received intensive research interest over the past decade because of its negative impact on socio-occupational outcome, quality of life and illness prognosis (1–3). However, it is unclear whether patients' cognitive deficits comprise a diagnostic intermediate phenotype that may aid diagnostic accuracy and represent a key treatment target. The present perspective paper evaluates the present evidence and discusses whether cognitive dysfunction in bipolar disorder qualifies as a diagnostic intermediate phenotype using the Robin and Guze criteria of diagnostic validity (4) also concurrent with the later endophenotype concept (5) and extended criteria suggestions (6). The rationale for the Robin and Guze criteria was to develop criteria distinguishing between various psychiatric disorders and aiming for a valid psychiatric classification system (4). An intermediate phenotype was later defined as a measurable component along the pathway between disease and distal genotype, and have emerged as an important concept in the study of complex neuropsychiatric diseases (5). An endophenotype may be neurophysiological, biochemical, endocrinological, neuroanatomical, cognitive, or neuropsychological in nature (5). The paper will review current data on cognitive dysfunction within (1) delineation of the clinical intermediate phenotype, (2) associations of the intermediate phenotype with para-clinical data such as brain imaging and blood-based data, (3) associations to family history / genetics, (4) characteristics during long-term follow-up, and (5) treatment effects on cognition. Within each of these five points, the specificity of the findings in relation to bipolar disorder compared with schizophrenia and unipolar disorder will be summarized. The paper will identify knowledge gaps and suggest recommendations for future research within each of the five areas.

## Delineation and characterization of the clinical intermediate phenotype

This area concerns whether cognitive dysfunction in bipolar disorder in remission is circumscribed clinically as a separate diagnostic intermediate phenotype of bipolar disorder and whether such an intermediate phenotype differs from similar intermediate phenotypes within related disorders such as schizophrenia and unipolar disorder.

Meta-analyses have consistently shown disturbances in executive function, verbal learning and memory, visual memory and attention in bipolar disorder compared with healthy control individuals (7–10). Cognitive impairment in the remitted phase of bipolar disorder is on average of a moderate effect size (7), however, with a substantial cognitive heterogeneity: 12–40% of patients present global cognitive impairments across several domains, 29–40% show selective deficits in attention and psychomotor speed, and 32–48% are relatively “cognitively intact” in comparison with norms (11). Subgroups with neurocognitive impairments present reduced functional capacity, more stress and poorer quality of life than patients who are cognitively intact, despite similar degrees of subsyndromal mood symptoms (2, 11, 12). Compared with bipolar disorder type II (hypomanic and depressive episodes; no manic episodes), bipolar disorder type I (manic and/or depressive episodes) seems to be associated with modestly more pronounced global cognitive impairment as well as increased disturbances in verbal memory, processing speed, executive function speed, and executive function accuracy (13).

On the other hand, cognitive deviances are not specific for bipolar disorder. Cognitive impairment is also prevalent in schizophrenia (14) and unipolar disorder (15), and there is no specific neuropsychological signature that can facilitate the diagnostic differentiation between bipolar disorder, schizophrenia, and unipolar disorder (16), notwithstanding, neuropsychological deficits appear more severe in schizophrenia (14, 17) and bipolar disorder (15). In schizophrenia and bipolar disorder, cognitive impairments have been found to correlate with socio-demographic (lower education and work capacity), clinical (more hospitalizations, longer duration of illness, negative psychotic symptoms, and non-remission status), treatment (antipsychotics, anti-cholinergics) variables and lower psychosocial functioning (1, 3, 18). Similar predictors of cognitive dysfunction are found in unipolar disorder but with more variable evidence, possibly because of the generally milder cognitive impairments in this patient group (19, 20).

Emotion dysregulation may be another cognitive feature of bipolar disorder that persists into periods of remission. Such deficits in “hot” (emotional) cognition are closely linked to emotional disturbances (21) and difficulties in socio-emotional behavior and interpersonal relations in bipolar disorder (22). Hot cognition abnormalities in bipolar disorder have been observed within three domains; emotional processing, reward processing, and emotion regulation [reviews in (23, 24)].

Emerging evidence points to partial persistence of such hot cognition dysfunction during remission in unipolar disorder, particularly within negative affect processing, (25) and the presence of similar abnormalities in healthy relatives of patients with unipolar disorder, at least at a neural level (25, 26). Hot cognition has not been systematically investigated across mood disorders and schizophrenia although some data point toward somewhat dissociable deficits in primary reward processing in unipolar disorder and schizophrenia (27). A key question remains whether deficits in experiencing rewards are independent of anhedonia in schizophrenia and whether level of observed reward disruption across unipolar disorder and schizophrenia a is a matter of severity rather than reflecting a qualitatively distinct mechanism (27). In contrast, a few studies of patients with bipolar disorder found evidence for a distinct positive bias in emotion processing and elevated reward responsiveness (28)—cognitive features that may in the future aid diagnostic discrimination between the disorders.

## Associations of the intermediate phenotype with para-clinical data such as brain imaging and blood-based data

It is unknown whether shared manifestations of cognitive dysfunction across diagnostic categories also reflect shared neurobiological mechanisms or whether the sources of impairment differ. A recent study investigated the associations between general cognitive deficits (non-emotional or so called “cold”) and functional network integrity measures including global and local efficiency of the whole brain, cingulo-opercular network (CON), frontoparietal network, and auditory network (29). Patients with schizophrenia and psychotic bipolar disorder had significantly reduced CON global efficiency compared with healthy controls (29). All patients with psychotic disorders had significantly reduced CON local efficiency, but the clinical groups did not differ from one another. The CON global efficiency was significantly associated with general cognitive ability across all groups and significantly mediated the association between psychotic disorder status and general cognition. It was concluded that these findings provide evidence that “reduced CON and subcortical network efficiency may play a role in the general cognitive deficit observed across the psychosis” (29).

Another common neural underpinning of cognitive deficits across bipolar disorder, unipolar disorder, and schizophrenia is aberrant task-related activity in the dorsal prefrontal cortex (PFC), although findings regarding the direction of the aberrant activity vary between studies with most evidence for *hypo-*activity in schizophrenia and bipolar disorder while the findings in unipolar disorder are more variable. In particular, we found in a systematic review of >100 neuroimaging studies across bipolar disorder and unipolar disorder consistent evidence for abnormal (predominantly hypo-) activity in dorsal and lateral PFC cognitive control regions during performance on working memory, executive skills, memory encoding, and sustained attention (Miskowiak and Petersen, in press). Notably, the *direction* of this dorsal PFC activity depended on patients' performance levels. Dorsal PFC *hypo*-activity is consistently linked to impaired task performance; that is *reduced cognitive capacity*. In contrast, dorsal PFC *hyper*-activity is generally accompanied by normal performance levels and thus seems to reflect *reduced cortical efficiency*; that is, a need to recruit more neural resources to maintain normal performance. These associations are likely to explain the more consistent evidence for dorsal PFC hypo-activity in the generally more severely cognitively impaired patients groups (i.e., schizophrenia and bipolar disorder).

Another consistent finding in the review was reduced deactivation of the default mode network (DMN) and limbic structures during active task performance across bipolar disorder and unipolar disorder (ibid). This suggests that cognitive impairments across mood disorders are exacerbated by a failure to suppress task-irrelevant neural activity associated with emotional reactivity, self-focus and rumination (ibid).

Emerging neuroimaging evidence points to deficits in emotion dysregulation being a prominent feature of bipolar disorder, while unipolar disorder seems to be more consistently associated with negative processing biases (30). Emotion dysregulation in bipolar disorder seems associated with increased activity in limbic regions implicated in emotion-generation paired with deficient lateral prefrontal top-down control of emotional responses (31). However, this finding is not specific to bipolar disorder; indeed neuroimaging studies of social cognition in patients with mood disorders have generally revealed enhanced activation in limbic and emotion-related structures and attenuated activity within frontal regions associated with emotion regulation and higher cognitive functions. These results reveal an “overall lack of inhibition by higher-order cognitive structures on limbic and emotion-related structures during social cognitive processing in patients with mood disorders” (32). Critically, key variables, including illness burden, symptom severity, comorbidity, medication status, and cognitive load may moderate this pattern of neural activation (32).

Peripheral inflammation might be related to cognitive deficits in schizophrenia and bipolar disorder. Single studies suggest the role of C-reactive protein (CRP), interleukin (IL)-1 receptor antagonist, IL-6, and tumor necrosis factor-α (TNF-α) with its receptors in the development of cognitive impairment in bipolar disorder as summarized in reviews (33, 34). Due to low number of studies, it is difficult to draw conclusions on the involvement of CRP and cytokine alterations in the development of cognitive deficits in bipolar disorder. More consistent results indicate worse cognitive performance in schizophrenia patients with higher CRP levels (33). Evidence for the involvement of other cytokines in cognitive impairment in patients with schizophrenia is less convincing due to discordant results and scarcity of studies (33). Nevertheless, a larger study found that general cognitive abilities may be associated with IL-1Ra and sTNF-R1 in schizophrenia and with soluble CD40 ligand (sCD40L) and IL-1Ra in bipolar disorder patients (35).

## Associations to family history / genetics

A recent meta-analysis of cognitive functions in first-degree relatives of probands with bipolar disorder and schizophrenia showed that probands with schizophrenia displayed cognitive deficits in all domains (d = 0.20–0.58) whereas probands with bipolar disorder underperformed healthy controls in processing speed, verbal fluency and speed based executive function tests (36). It was concluded that “inefficiency in processing information and impaired processing speed might be common vulnerability factors for major psychoses.” On the other hand, “low performance in accuracy based tasks and deficits in general intellectual ability, verbal learning, planning, and working memory might be more specifically associated with risk for schizophrenia” (36). Further, we found in a systematic review of neuroimaging studies of healthy first-degree relatives of patients with bipolar disorder emerging evidence for abnormalities in emotional processing—and regulation and reward processing being candidate endophenotypes (37). We investigated this notion in a cohort of monozygotic twins at risk of either unipolar or bipolar disorder (reflected by a co-twin history of that disorder) (38). Interestingly, we found that twins at risk of bipolar disorder showed increased sensitivity and reactivity to positive social stimuli in comparison with individuals at risk of unipolar disorder and low-risk control twins. Together, these findings provide emerging evidence for positive bias being a putative neurocognitive endophenotype that is specific for bipolar disorder.

In terms of neurocognitive-genetic investigations, catechol-O-methyltransferase (COMT) and brain-derived neurotrophic factor (BDNF) are the two most studied candidate genes especially in patients with schizophrenia (39). Whereas BDNF Val66Met carriers seem to perform worse on verbal working memory, problem solving, and visuo-spatial abilities, COMT Val158Met carriers may perform better in working memory, attention, executive functioning with evidence of genotype by diagnosis interactions including high-risk individuals (39), although findings are not uniform (40, 41). In terms of genetic-structural MRI studies, “patients with schizophrenia are found to have reductions in the frontal, temporal, parietal cortices, and limbic regions, which are associated with BDNF, COMT, and neuregulin-1 (NRG1) genes” (39). Genetic-functional MRI studies in bipolar disorder are sparse and results conflicting (39, 42).

## Characteristics of cognition during long-term follow-up

Cognitive deficits in bipolar disorder in remission seem to persist over time or even progress supporting the view that these deficits qualify for an intermediary phenotype. Using a 5 years longitudinal cohort, 91 individuals with bipolar disorder and 17 healthy controls were administered a battery of neuropsychological tests that captured four main areas of executive functioning that were found to persist over time (43).

Based on cross-sectional studies, cognitive deficits seem to deteriorate during late stages of the disorder (44). In contrast, there is a lack of longitudinal studies on cognition in bipolar disorder (45, 46) with the largest study being the study by Ryan et al. (43, 47). A new meta-analysis comparing short-term (mean of 1.5 years) and long-term (mean of 5.5 years) neurocognitive changes in 643 euthymic patients with bipolar disorder, 367 healthy controls and 168 patients with schizophrenia found no cognitive changes over time in any of the three cohorts (46). Besides the small sample sizes in each study, limitations included short follow-up (mean follow-up period of 4.6 years) specifically for studies of bipolar disorder, high attrition rates (up to 45%) among all participants and strict euthymia criteria for bipolar patients included in the analyses, which may have introduced a selection bias (including only the high functioning patients), as also concluded in a prior similar meta-analysis of bipolar disorder (48).

Regarding cognitive functioning in unipolar disorder, some cross-sectional studies suggest that cognitive function in the euthymic phase is associated with the duration or number of prior episodes [(49–54), for a review see: (19)].

Studies on the risk of developing dementia in unipolar disorder and bipolar disorder have recently been summarized (55). It was concluded that a meta-analysis including 44 studies on depression and six on bipolar disorder (56) as well as *all* subsequent studies have confirmed that unipolar disorder (56–60) and bipolar disorder (56–58, 60, 61) are associated with increased risks of developing dementia long-term (as a clinical diagnosis). It was further concluded that longitudinal studies of bipolar disorder may have had to short follow-up time (mean follow-up period of 4.62 years) to reveal a decrease in *neuropsychological* functioning over time in contrast to the much longer follow-up time in studies with dementia as the outcome measure (55).

## Treatment effects on cognition

A recent systematic review on novel pharmacological (N-acetyl cysteine, pregnolone, ketamine and pramipexole, mifepristone, galantamine, insulin, erytrophoietin, withania somnifera, and citicoline) and psychological treatments (cognitive remediation and cognitive training) on cognition in bipolar disorder identified 19 studies of which 13 were RCTs and six were open-label or non-randomized studies (62). The efficacy on cognition was overall disappointing or preliminary, possibly due to several methodological challenges. Similarly, a later controlled trial found no effect of methylene blue on cognition in bipolar disorder (63). Among the most promising pharmacological treatments for cognitive dysfunction across bipolar disorder and unipolar disorder is erythropoietin, but the evidence is still preliminary (62, 64). These findings are partly in accordance with findings within unipolar disorder and schizophrenia with only a few studies have shown benefit for pharmacological treatments (64–66) and with a lack of successful replication of these data (64, 66, 67). However, psychological treatment programs involving intensive cognitive remediation have revealed more consistent positive effects on cognition in schizophrenia (68, 69) and emerging evidence in mood disorders (64, 70).

## Conclusion and recommendations for future research

It is clear from the present summary of studies on cognition in bipolar disorder that at the current state of knowledge cognition in bipolar disorder does not qualify as a diagnostic intermediate phenotype using the Robin and Guze criteria of diagnostic validity (4) or the later endophenotype concept (5, 6), although emerging evidence points to hot cognition abnormalities representing promising putative endophenotypes. Rather, extant findings within four of the five Robin and Guze criteria generally support the dimensional hypothesis that a shared neurobiological mechanism underlies cognitive impairment across bipolar disorder, unipolar disorder and schizophrenia: (1) there may not be a specific neuropsychological signature that differentiate cognitive deviances in bipolar disorder from those in schizophrenia and unipolar disorder (only potentially within hot cognition); (2) brain imaging or blood-based data does not at the current state of knowledge differentiate between cognitive dysfunction in bipolar disorder, schizophrenia or unipolar disorder; (3) probands to patients with bipolar disorder, schizophrenia, and unipolar disorder show similar cognitive deficits although with varying severity, except for within hot cognition. Investigations of genetic associations to cognitive deviances are in its early stages, only (4) treatment effects of pharmacological or psychological interventions on cognition do not seem to differ within bipolar disorder, schizophrenia and unipolar disorder. The fourth Robin and Guze criterion seems fulfilled as cognitive deficits in bipolar disorder seem either stable over time or progress during long-term supporting cognitive deficits as an intermediary phenotype.

It is further evident from the present summary of studies on cognition in bipolar disorder that a number of research initiatives are needed within all five of the Robin and Guze criteria.

1. Research is needed integrating “hot” and “cold” cognition in bipolar disorder. Few if any studies have investigated how emotion dysregulation (i.e., hot cognition) interact with cold cognition. As recently emphasized, cognitive biases, reward processing and motivation, rumination, and mood stability may play significant roles in the manner in which attention, appraisal, and response processes are deployed in mood disorders (71).

2. Emotion dysregulation (hot cognition) should be investigated across mood disorders and schizophrenia. Emotion dysregulation has emerged as a new research area that may characterize mood disorders, and potentially specifically bipolar disorder, rather than schizophrenia. Although these speculations are clinically plausible, emotion dysregulation has not been systematically investigated across mood disorders and schizophrenia.

3. Structural and functional neuroimaging data on cognitive features (“cold” and “hot”) should be integrated across mood disorders. Such multimodal neuroimaging studies aiming to identify structure-function relationships in neural circuitry have previously been suggested in relation to bipolar disorder in general (24). As highlighted, a very small number of studies examined structure-function relationships in prefrontal cortical-amygdala circuitry in adults with bipolar disorder type I and bipolar disorder type II (24). We suggest integrating investigations of “cold” and “hot” cognitive features into the loop and across mood disorders.

4. Neurogenetics should be integrated into research in cognitive disturbances in patients with mood disorders and schizophrenia and in their first degree relatives.

5. Research in cognitive enhancement treatments. We have previously suggested implementation of a ‘neurocircuitry-based’ biomarker model to evaluate neural target engagement in cognitive enhancement (62). We suggest that a valid biomarker model for cognitive improvement must fulfill five key validity criteria: it must (i) be sensitive to a treatment with pro-cognitive effects, (ii) produce similar effects in patients with cognitive dysfunction and healthy participants, (iii) be sensitive to effective treatments with different neurochemical mechanisms, (iv) be unresponsive to ineffective treatments, and (v) be sensitive to both cognitive improvement and—decline. A potential solution to the problem is a step-wise approach with which we: (i) identify the most reliable functional neuronal correlates of cognitive deficits in neuropsychiatric disorders, (ii) select one of the most promising candidate treatments and test its ability to modulate the activity in these dysfunctional neural circuitries in a short-term proof-of-concept fMRI study, and (iii) if target engagement is shown in (ii), then test the effects of this candidate treatment in a longer-term clinical phase 2 trial in patients using fMRI to elucidate the neuronal changes underlying potential pro-cognitive effects.

More evidence is needed confirming whether cognitive deficits comprise a diagnostic intermediate phenotype in bipolar disorder. The long-term perspective is that cognitive deficits may aid diagnostic accuracy and represent a key treatment target in bipolar disorder.

## Author contributions

LK developed the idea and drafted the first version of the paper. KM revised the paper and both authors accepted the final version of the paper.

### Conflict of interest statement

LK has received consultancy fees from Sunovion in the past 3 years. KM has received consultancy fees from Lundbeck and Allergan in the past 3 years.
